# The roles and mechanism of IFIT5 in bladder cancer epithelial–mesenchymal transition and progression

**DOI:** 10.1038/s41419-019-1669-z

**Published:** 2019-06-04

**Authors:** Jun Huang, U-Ging Lo, Shiqi Wu, Bin Wang, Rey-Chen Pong, Chih-Ho Lai, Ho Lin, Dalin He, Jer-Tsong Hsieh, Kaijie Wu

**Affiliations:** 1 Department of Urology, First Affiliated Hospital of Xi’an Jiaotong University, Xi’an, P.R. China; 2 Department of Urology, The Second Xiangya Hospital, Central South University, Changsha, P.R. China; 3 Department of Urology, University of Texas Southwestern Medical Center, Dallas, TX USA; 4 Department of Microbiology and Immunology, Graduate Institute of Biomedical Sciences, College of Medicine, Chang Gung University, Taoyuan, Taiwan; 5 Department of Life Sciences, National Chung Hsing University, Taichung, Taiwan; 6 Department of Biotechnology, Kaohsiung Medical University, Kaohsiung, Taiwan

**Keywords:** Bladder cancer, miRNAs

## Abstract

The prognosis of bladder cancer (BCa) depends on several key factors including anatomical site, tumor grade, and stage. In general, muscle-invasive bladder cancer (MIBC) is associated with higher incidence of distant metastasis compared with Non-muscle-invasive bladder cancer (NMIBC). Treatment outcome of the patients with metastatic BCa has been very poor with ~15% of overall survival rate. Thus, it is apparently important to understand the underlying biology for metastatic progression of BCa. Although epithelial–mesenchymal transition (EMT) has long been implicated in BCa metastasis and treatment resistance, the underlying mechanism is not fully understood. In this study, we have identified that the expression of interferon induced protein with tetratricopeptide repeats 5 (IFIT5) is positively correlated with pathological characteristics, and predicts a poor prognosis of BCa patients. Since the function of IFIT5 in BCa has not yet been characterized, we demonstrate that IFIT5 can induce EMT, promote cell migration and invasion, and increase the expression of ICAM1 in BCa via down-regulation of mature miR-99a. Moreover, ICAM1 is shown as a direct target of miR-99a. Overall, we conclude that IFIT5 is a new oncogene in BCa.

## Introduction

In USA, bladder cancer (BCa) is the second common urological cancer in men, it was estimated that there was 60,490 new bladder cancer cases in men among 2017^1^. More than 70% of BCa are non-muscle-invasive bladder cancer (NMIBC), transurethral resection together with intravesical bacillus Calmette-Guérin (BCG) instillation is one of the most common therapeutic strategies for NMIBC, even though, more than 50% of patients will have disease relapse, 10–30% NMIBC will progress to muscle-invasive bladder cancer (MIBC), and even metastatic bladder cancer^2^, but the specific mechanism is still unknown.

Epithelial–mesenchymal transition (EMT) is a multistep process in which epithelial cells lose their epithelial characteristics and gain mesenchymal characteristics. Numerous in vitro and in vivo studies suggest that EMT is an initial event associated with cancer cell invasion and metastasis in various malignancies, including BCa^3^. Our previous studies in prostate cancer and renal carcinoma showed that a interferon induced gene interferon induced protein with tetratricopeptide repeats 5 (IFIT5) acted as an oncogenic gene via promoting EMT^4,5^, but the role of IFIT5 in bladder cancer is still unknown. In this study, we have identified a new regulatory pathway mediated by IFIT5, which is able to process the stability of suppressor microRNA (miR) in bladder cancer. Clinically, the expression of IFIT5 is positively correlated with the pathological characteristic of BCa, but negatively correlated with BCa patient survival. Apparently, elevated IFIT5 is able to increase in vivo tumor growth as well as EMT associated with in vitro cell migration and invasiveness. Mechanistically, IFIT5 is able to regulate miR-99a maturation that can target ICAM1-elicited EMT pathway. Taken together, we conclude that IFIT5 represents a new oncogene in BCa with unique mechanism of action in modulating tumor suppressor miR.

## Results

### Expression of IFIT5 in BCa is positively correlated with the pathological characteristics, but negatively correlated with patient survival

**Fig. 1:**
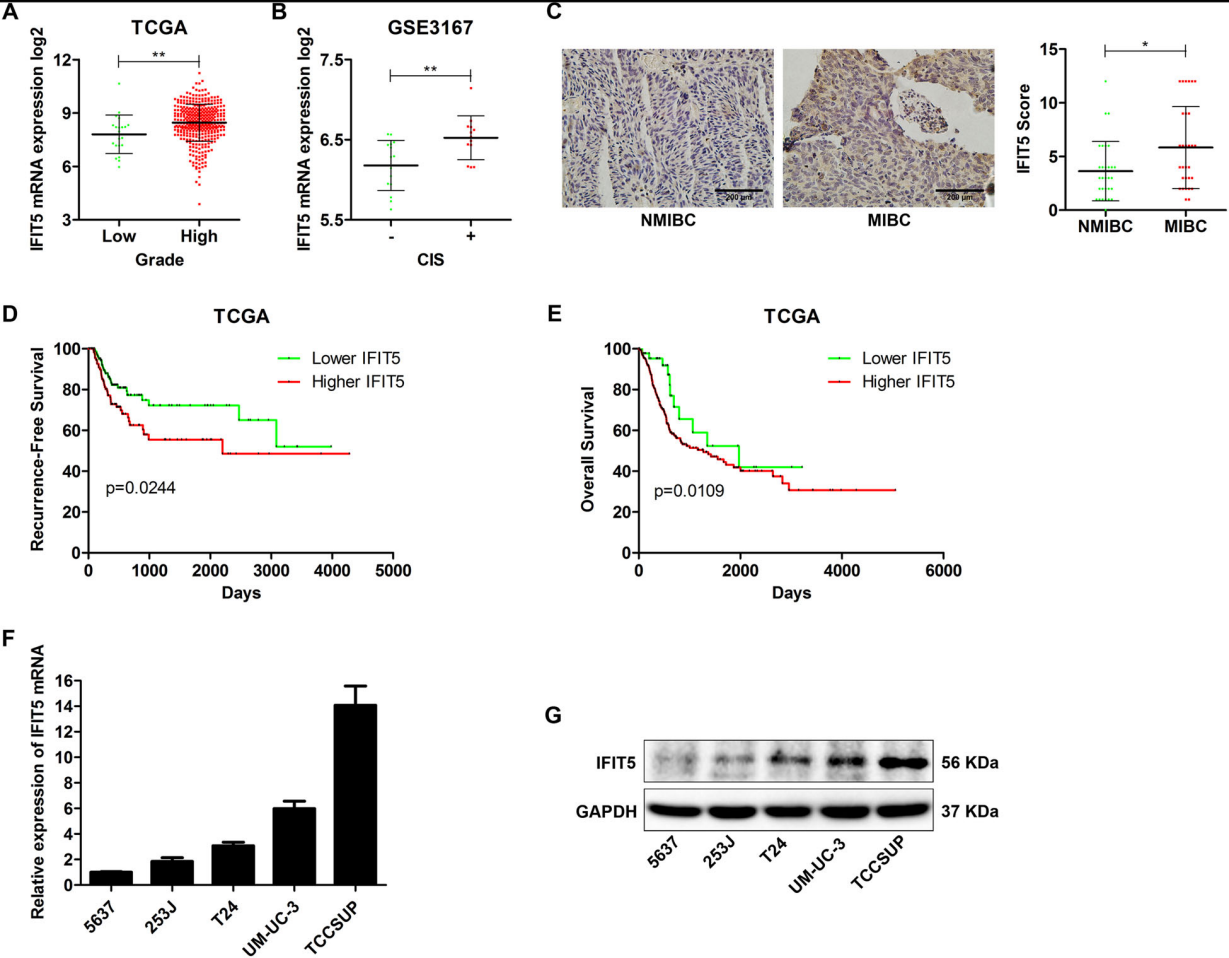
Expression of IFIT5 is positively correlated with BCa pathological characteristics, and negatively correlated with patient survival **a** Expression of IFIT5 in high-grade BCa tissues is higher than that in low-grade BCa tissues. **b** Expression of IFIT5 in CIS-positive NMIBC tissues is higher than that in CIS-negative NMIBC tissues. **c** Expression of IFIT5 in NMIBC tissues is lower than that in MIBC tissues. **d**, **e** Higher expression of IFIT5 in BCa tissue correlated with worse patient recurrence-free survival and overall survival. **f**, **g** Relative expression of IFIT5 mRNA and protein in BCa cell lines 5637, 253 J, T24, UM-UC-3 and TCCSUP. *P < 0.05, **P < 0.01

To investigate the expression level of IFIT5 in BCa tissues, we retrieved TCGA and GEO database to analyze the association between IFIT5 expression and the pathological characteristics of BCa. We found that the expression of IFIT5 mRNA was higher in high-grade BCa tissues (Fig. 1a) or NMIBC tissues with carcinoma in situ (CIS) (Fig. 1b). Immunohistochemical staining of IFIT5 expression between NMIBC and MIBC tissues indicated that IFIT5 expression in MIBC tissues was higher than in NMIBC tissues (Fig. 1c). Then, based on the TCGA data, we analyzed the correlation between IFIT5 expression and BCa patients’ recurrence-free survival (RFS) and overall survival (OS), and the results showed that the patients with higher IFIT5 expression would have a shorter RFS and OS (Fig. 1d, e). Also, by profiling IFIT5 mRNA and protein expression in several BCa cell lines, 5637 derived from grade II BCa exhibited the lowest IFIT5 mRNA and protein expression, while TCCSUP derived from grade IV BCa exhibited the highest IFIT5 mRNA and protein expression (Fig. 1f, g).

### IFIT5 induces BCa EMT, promotes cell migration and invasion in vitro

**Fig. 2:**
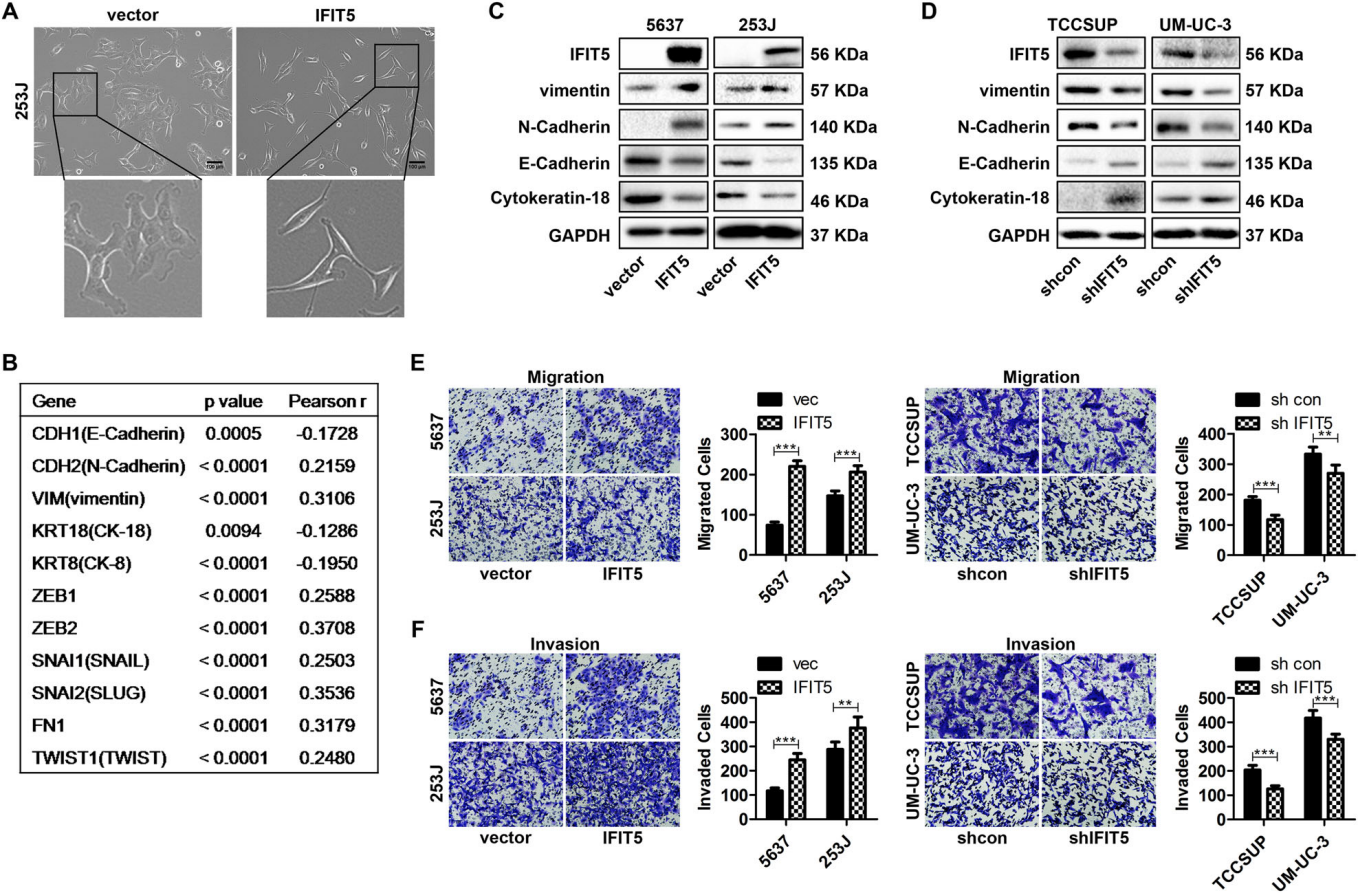
IFIT5 induces EMT to promote cell migration and invasion of BCa cells. **a** Morphological changes of 253 J cells with OE of IFIT5. **b** TCGA data shows the expression correlation between IFIT5 and EMT-associated genes in BCa tissues. **c** OE of IFIT5 in 5637 and 253 J cells induces EMT. **d** KD of IFIT5 in TCCSUP and UM-UC-3 cells inhibits EMT. **e** OE of IFIT5 in 5637 and 253 J promotes cell migration, while KD of IFIT5 in TCCSUP and UM-UC-3 inhibits cell migration. **f** OE of IFIT5 in 5637 and 253 J promotes cell invasion, while KD of IFIT5 in TCCSUP and UM-UC-3 inhibits cell invasion. **P < 0.01, ***P < 0.001

To study the function of IFIT5 in BCa, we generated several stable BCa cell sublines with over-expression (OE) or knocked-down (KD) of IFIT5. Noticeably, we found that the morphology of IFIT5-OE in 253 J cells gained mesenchymal features with spindle-like and more scattered shape (Fig. 2a), suggesting that IFIT5 could increase EMT in BCa cells. Thus, we analyzed the expression correlation between IFIT5 and EMT-associated genes in BCa tissues from TCGA database; indeed, the expression of IFIT5 was negatively correlated with CDH1 (E-Cadherin), KRT8 (Cytokeratin-8, CK-8), and KRT18 (Cytokeratin-18, CK-18), but positively correlated with CDH2 (N-Cadherin), VIM (vimentin), ZEB1, ZEB2, SNAI1 (SNAIL), SNAI2 (Slug), FN1, and TWIST1 (Fig. 2b). Furthermore, we confirmed that OE of IFIT5 in 5637 and 253 J cells could increase vimentin and N-Cadherin, but decrease E-Cadherin and Cytokeratin-18 (Fig. 2c); meanwhile, KD of IFIT5 in TCCSUP and UM-UC-3 cells could decrease vimentin and N-Cadherin, but increased E-Cadherin and Cytokeratin-18 (Fig. 2d). These data supported that IFIT5 could induce EMT in BCa cells.

Functionally, we employed Transwell assay to investigate the effect of IFIT5 on cell migration and invasion in BCa. As shown in Fig. 2e, f, OE of IFIT5 in 5637 and 253 J promoted cell migration and invasion, in contrast, KD of IFIT5 in TCCSUP and UM-UC-3 inhibited cell migration and invasion. However, neither OE nor KD of IFIT5 in BCa cells significantly change cell proliferation of these BCa cells based on MTT assays (Supplementary Fig. 1).

### IFIT5 negatively regulates the expression of mature miR-99a in BCa cells

**Fig. 3:**
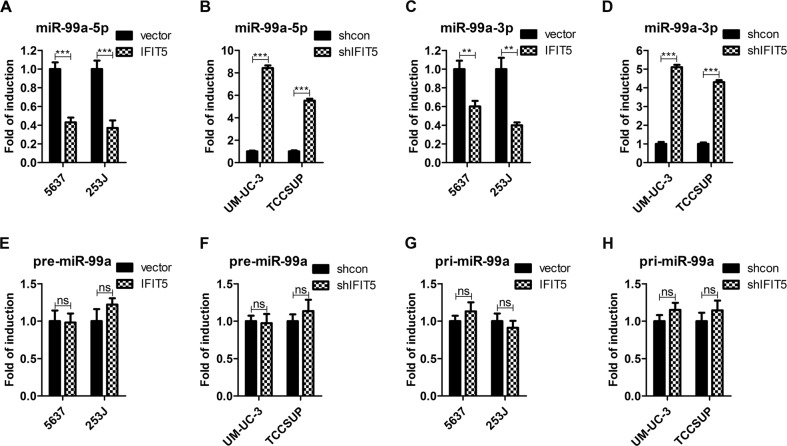
IFIT5 decreases the expression of mature miR-99a in BCa cells. **a** OE of IFIT5 in 5637 and 253 J cells decreases miR-99a-5p expression. **b** KD of IFIT5 in TCCSUP and UM-UC-3 cells increases miR-99a-5p expression. **c** OE of IFIT5 in 5637 and 253 J cells decreases miR-99a-3p expression. **d** KD of IFIT5 in TCCSUP and UM-UC-3 cells increases miR-99a-3p expression. **e**, **f** Neither OE nor KD of IFIT5 in 5637 or 253 J cells affects pre-miR-99a expression. **g**, **h** Neither OE nor KD of IFIT5 in TCCSUP or UM-UC-3 cells affects pri-miR-99a expression. **P < 0.01, ***P < 0.001, ns: no significance

Our previous study in prostate cancer demonstrated that IFIT5 could negatively regulate the maturation of pre-miR-363, pre-miR-101, and pre-miR-128 that can target EMT transcription factors such as Slug and ZEB1^4^. Whether the similar effect of IFIT5 on BCa remained undetermined, thus, we applied microRNA PCR array to screen microRNAs that may be negatively regulated by IFIT5 in BCa cells. As compared to the control cells, few microRNAs were up-regulated in IFIT5-KD UM-UC-3 cells (Supplementary Table 1). Among these microRNAs, miR-99a-5p was the most potential one that may be up-regulated in IFIT5-KD cells. Subsequently, qRT-PCR was employed to validate that OE of IFIT5 in 5637 and 253 J cells could decrease miR-99a-5p (Fig. 3a) while KD of IFIT5 in UM-UC-3 and TCCSUP cells could increase miR-99a-5p (Fig. 3b). Similarly, IFIT5 could also negatively regulate the expression of miR-99a-3p in BCa cells (Fig. 3c, d). On the other hand, the presence of IFIT5 had no effect on the expression level of pre-miR-99a and pri-miR-99a in BCa (Fig. 3e–h). In addition, we have applied qRT-PCR to detect the expression of miR-363, miR-101, and miR-128 in IFIT5 OE or KD BCa cells, the results showed that the expression change of miR-363, miR-101, and miR-128 were not as significant as miR-99a (Supplementary Fig. 2). Taken together, we believe that the mechanism of action of IFIT5 is to inhibit the expression of mature miR-99a in BCa.

### miR-99a antagonizes the IFIT5-elicited EMT associated with cell migration and invasion in BCa

**Fig. 4:**
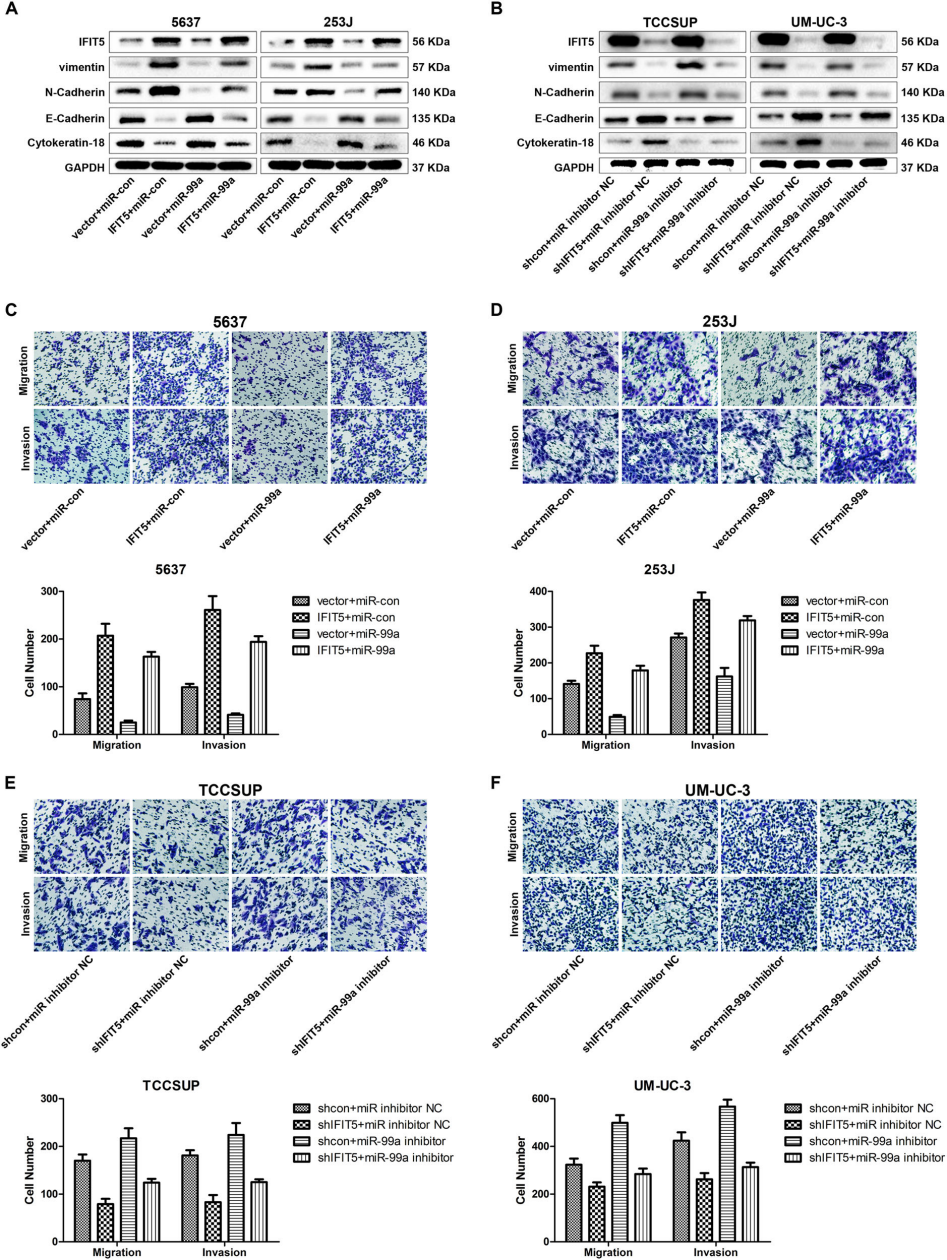
miR-99a suppresses the effect of IFIT5 on EMT, cell migration and invasion of BCa. **a** miR-99a partially reverses the effects of IFIT5 on EMT of 5637 and 253 J cells. **b** miR-99a inhibitor partially reverses the effects of IFIT5-KD on EMT of TCCSUP and UM-UC-3 cells. **c**, **d** miR-99a partially reverses the effects of IFIT5 on cell migration and invasion of 5637 and 253 J cells. **e**, **f** miR-99a inhibitor partially reverses the effects of IFIT5-KD on cell migration and invasion of TCCSUP and UM-UC-3 cells

To investigate the effect of miR-99a on IFIT5-elicited EMT, cell migration and invasion in BCa, the ectopic expression of pre-miR-99a in both IFIT5-OE 5637 and 253 J cells was generated. Western blot analysis indicated that miR-99a could diminish the IFIT5-elicited vimentin and N-Cadherin expression and restore the expression of E-Cadherin and Cytokeratin-18 in IFIT5-OE 5637 and 253 J cells (Fig. 4a). Similarly, miR-99a-3p inhibitor could diminish the expression of E-Cadherin and Cytokeratin-18 but restore the expression of vimentin and N-Cadherin in both IFIT5-KD TCCSUP and UM-UC-3 cells (Fig. 4b) Consistently, Transwell assay indicated IFIT5-induced cell migration and invasion were abolished by elevated miR-99a in IFIT5-OE 5637 and 253 J cells (Fig. 4c, d). Meanwhile, the inhibition of cell migration and invasion induced by KD of IFIT5 were partially restored by transfection of miR-99a-3p inhibitor in TCCSUP and UM-UC-3 cells (Fig. 4e, f). These data support the suppressive functional role of miR-99a in IFIT5-induced EMT leading to cell migration and invasion in BCa.

### miR-99a targets ICAM1 mRNA induced by IFIT5 in BCa

**Fig. 5:**
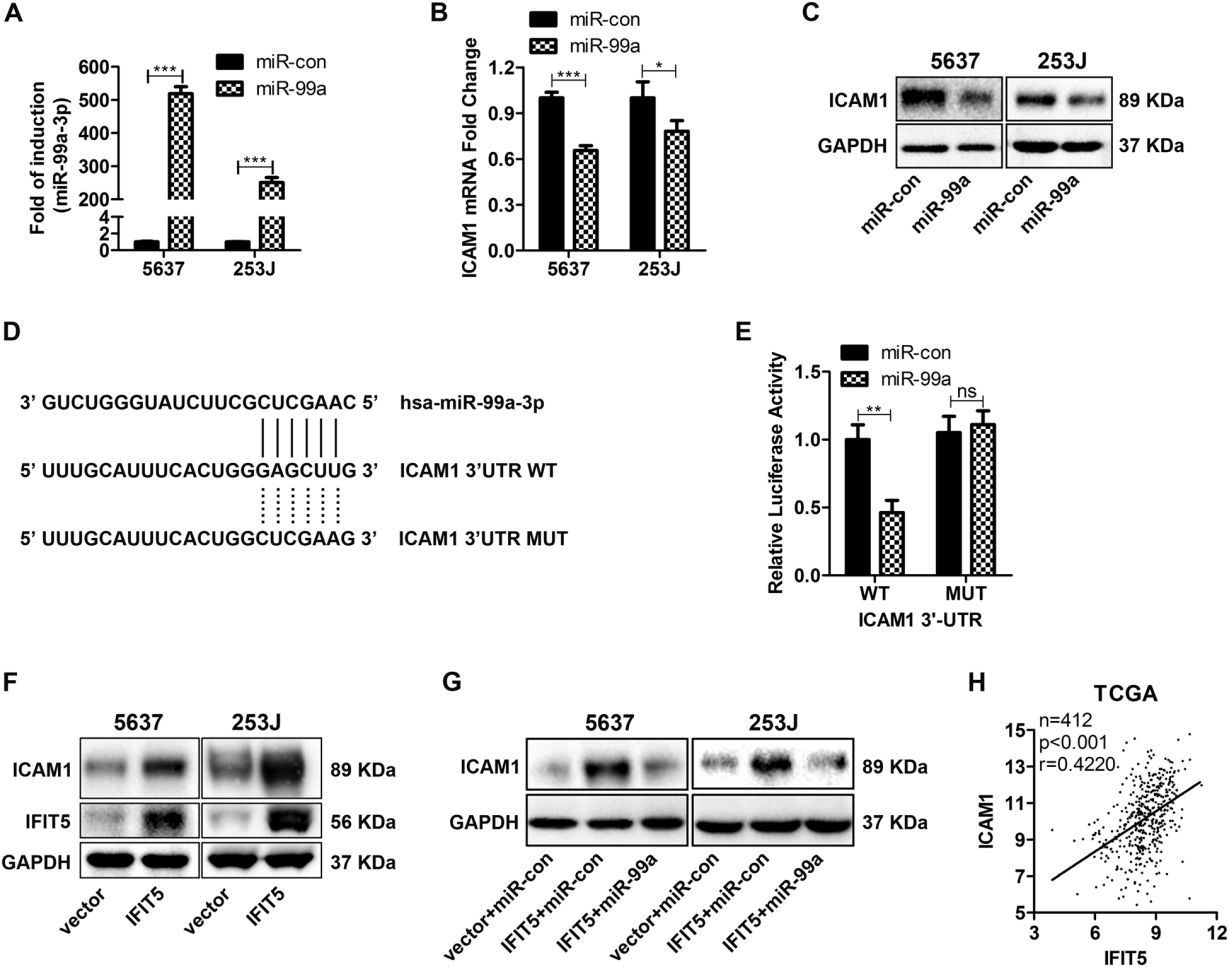
ICAM1 is a direct target of miR-99a-3p and IFIT5 increases ICAM1 via down-regulation of mature miR-99a in BCa. **a** Transfection of pre-miR-99a expression plasmid increases miR-99a-3p significantly in 5637 and 253 J cells. **b**, **c** miR-99a decreases ICAM1 mRNA and protein in 5637 and 253 J. **d** Schematic diagram of wild type (WT) and mutant type (MUT) of ICAM1 3′-UTR. **e** Dual luciferase assay in 293 T cells shows miR-99a deceases the relative luciferase activity in WT ICAM1 3′-UTR, but not MUT ICAM1 3′-UTR. **f** OE of IFIT5 increases ICAM1 expression in 5637 and 253 J cells. **g** miR-99a reverses the effects of IFIT5 on ICAM1 expression in 5637 and 253 J. **h** TCGA data shows a positive correlation between IFIT5 and ICAM1 in BCa tissues. *P < 0.05, **P < 0.01. ***P < 0.001, ns: no significance

The most common mechanism for microRNA is to degrade target mRNA by binding to its 3′ un-translated region (3′-UTR) or/and inhibit its translation. By screening microRNA.org (http://www.microrna.org/microrna/home.do), we identified ICAM1 (intercellular adhesion molecule 1) as a potential target gene of miR-99a-3p. Indeed, ectopic expression of miR-99a in 5637 and 253 J cells (Fig. 5a) could significantly decrease both ICAM1 mRNA and protein expression (Fig. 5b, c). Using wild type (WT) and mutant (MUT) 3′-UTR reporter constructs (Fig. 5d), miR-99a-3p could only decrease WT luciferase activity but not MUT (Fig. 5e), supporting that ICAM1 is a direct target of miR-99a in BCa.

A recent study indicates that IFIT5 could increase ICAM1 via activation of NF-kB in 293 and Hela cells^6^. Indeed, we found that ICAM1 protein could be induced in IFIT5-OE 5637 and 253J cells (Fig. 5f) and the elevated ICAM1 could be abolished after transfection of miR-99a (Fig. 5g). In addition, there is a positive correlation between IFIT5 and ICAM1 mRNA expression in BCa tissues from TCGA database (Fig. 5h). Taken together, these data suggest that ICAM1, a direct target gene of miR-99a, is involved in ITIF5-induced EMT.

### ICAM1 is involved in EMT induction by IFIT5 in BCa

**Fig. 6:**
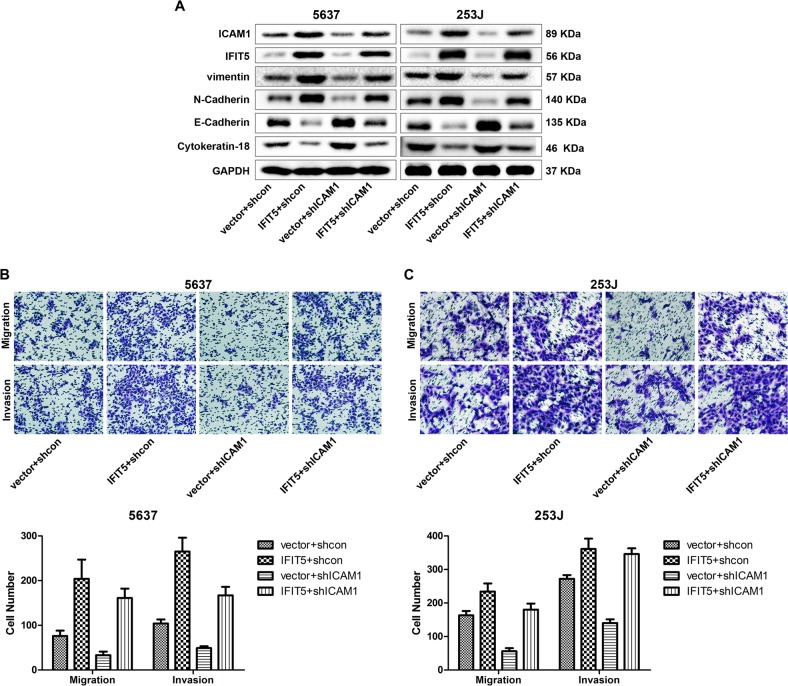
ICAM1 is involved in EMT induction by IFIT5 in BCa. **a** KD of ICAM1 partially suppresses EMT induced by IFIT5-OE in 5637 and 253 J cells. **b, c** KD of ICAM1 partially suppresses cell migration and invasion in IFIT5-OE 5637 and 253 J cells

To explore whether ICAM1 is involved in IFIT5-induced EMT, cell migration and invasion, we knocked-down ICAM1 expression in IFIT5-OE 5637 and 253J cells, and applied western blot and transwell assays to detect the expression change of EMT marker and ability of cell migration and invasion. Our data showed that KD of ICAM1 could partially down-regulate vimentin and N-Cadherin expression but restore E-Cadherin and Cytokeratin-18 expression induced by IFIT5 (Fig. 6a); meanwhile cell migration and invasion were also suppressed by KD of ICAM1 in IFIT5-OE 5637 and 253 J cells (Fig. 6b, c). Taken together, upregulation of ICAM1 is involved in EMT induction, cell migration, and invasion in IFIT5-OE BCa cells.

### IFIT5 promotes in vivo tumorigenesis, decreases the expression of mature miR-99a and induces EMT in BCa

**Fig. 7:**
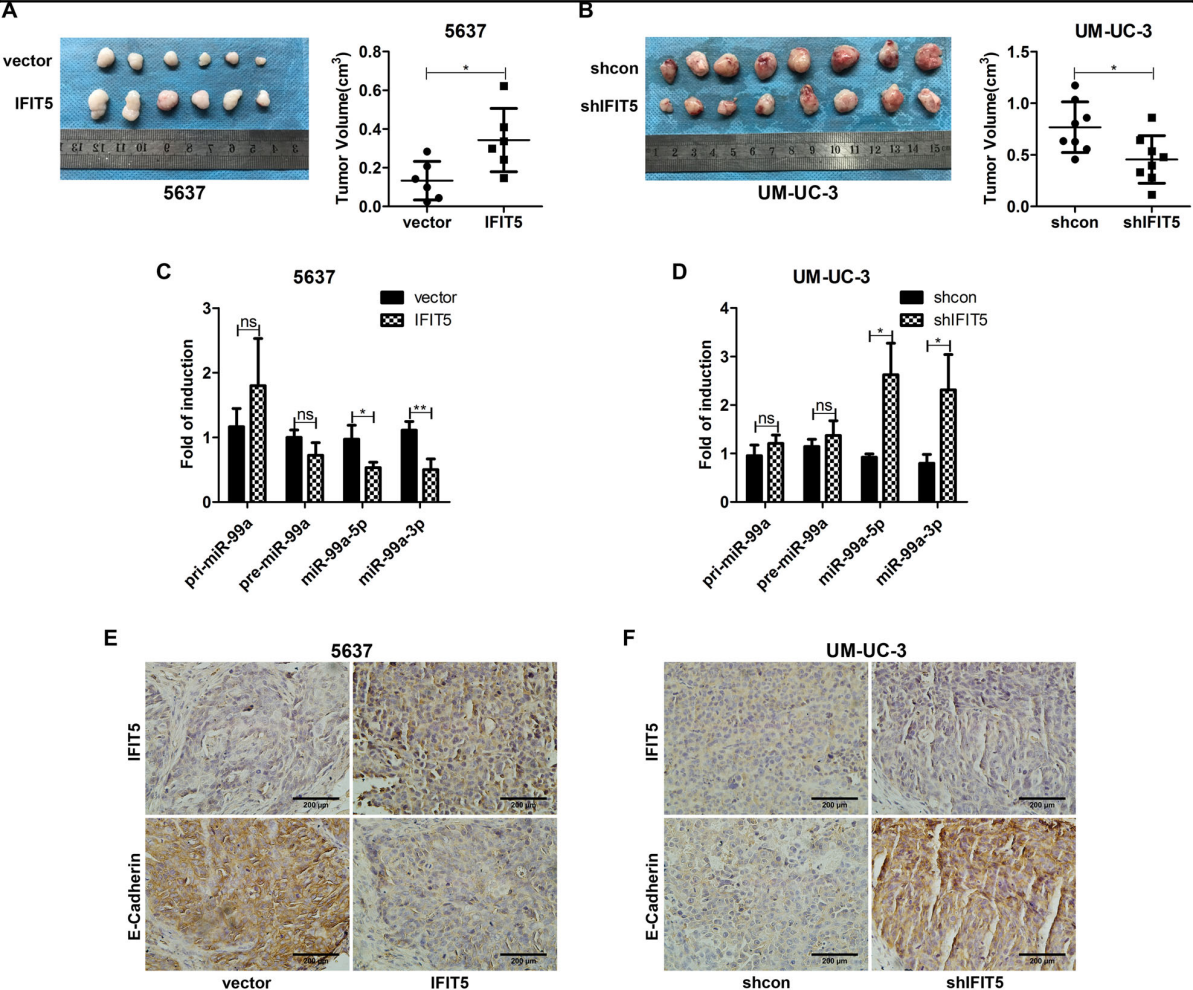
IFIT5 promotes BCa tumorigenesis and EMT and decreases the expression of mature miR-99a in vivo. **a** OE of IFIT5 in 5637 promotes tumorigenesis in vivo. **b** KD of IFIT5 in UM-UC-3 inhibits tumorigenesis in vivo. **c** OE of IFIT5 decreases the expression of mature miR-99a, but has no effects on the expression of pre-miR-99a and pri-miR-99a in vivo. **d** KD of IFIT5 increases the expression of mature miR-99a, but has no effects on the expression of pre-miR-99a and pri-miR-99a in vivo. **e** OE of IFIT5 decreases the expression of E-Cadherin in subcutaneous xenografts. **f** KD of IFIT5 increases the expression of E-Cadherin in subcutaneous xenografts. *P < 0.05, **P < 0.01, ns: no significance

To validate our in vitro data about the effect of IFIT5, we generated subcutaneous xenograft model by injection of 5637 or UM-UC-3 subline cells into nude mice. Two weeks after injection, the tumor volume of IFIT5-OE 5637 xenografts was significantly higher than the controls, in contrast, the tumor volume of IFIT5-KD UM-UC-3 xenografts was significantly smaller than the controls (Fig. 7a, b), indicating that IFIT5 could promote BCa development. Using these xenograft samples, we were able to validate the expression profile of pri-miR-99a, pre-miR-99a, and mature miR-99a; the expression of mature miR-99a-5p and miR-99a-3p was decreased in IFIT5-OE xenografts, but increased in IFIT5-KD xenografts. On the other hand, neither the expression of pri-miR-99a nor pre-miR-99a was affected by IFIT5 in these xenografts (Fig. 7c, d). In addition, we used IHC staining to confirm the loss of E-Cadherin expression in IFIT5-OE xenografts, but the presence of E-Cadherin expression in IFIT5-KD xenografts (Fig. 7e, f) to support the according status of EMT in these different xenografts. Consistently, ICAM1 expression was increased in IFIT5-OE 5637 xenografts but decreased in IFIT5-KD UM-UC-3 xenografts (Supplementary Fig. 3).

## Discussion

Our recent data unveil that IFIT5 is a novel oncogene in prostate cancer by eliciting EMT via miR processing^4^. However, the role of IFIT5 in BCa has not been examined. Clinically, the expression of IFIT5 in BCa was positively correlated with pathological characteristics, and negatively correlated with the prognosis of BCa patients. However, we failed to observe that concomitant high IFIT5/low pre-miR-99a expression better stratified patients’ survival (Supplementary Fig. 4), because no expression data of mature miR-99a was available in TCGA database. Similarly, IFIT5 is able to induce EMT in BCa as shown in PCa^4^. However, in BCa, the mechanism of action of IFIT5 is to suppress miR-99a maturation but not miR-363, −128 and −101 in PCa, suggesting that IFIT5 represents a common miR processing machinery in various cell types with different miR contexts. Collectively, this study is the first time to unveil the oncogenic role and mechanism of IFIT5 in BCa and the outcome of this study could provide a new therapeutic target for reducing BCa metastases.

All IFIT proteins have tetratricopeptide repeats (TPRs), which are 34-amino acid, degenerate helix-turn-helix motif. TPRs usually present in multiple copies as tandem array and generate solenoid-type scaffolds, which are well suitable for mediating protein-protein or protein-RNA interaction^7^. As its special protein structure and the enriched positively charged residues at the inner protein surface^8^, most studies about IFIT family members were focus on IFIT1-IFIT2 and IFIT3 complex; and their roles in antivirus defenses and immune regulation^9–11^. A few studies described their potential role in cancer progression as a tumor suppressor. For example, in hepatocellular carcinoma, STAT1-STAT2 heterodimerization and nuclear translocation were enhanced by IFN-α-induced IFIT3, which enhanced the antitumor effects of IFN-α in turn^12^. In lymphoma cells, IFIT3 was reported to inhibit cell proliferation through upregulation of p21 and p27^13^. In oral squamous cell carcinoma, cell migration ability can be reduced by IFN-β-induced IFIT2, and better patient survival was associated with higher IFIT2 protein in tumor tissues^14^. IFIT5 is somehow different from other IFIT family members; it only exits as oligomers and human IFIT5 has no murine ortholog. Now, our recent study and this study demonstrate that IFIT5 can induce EMT and promote cell migration as an oncogene, which is totally different with IFIT2 and IFIT3.

IFIT5 is acting as a RNA-binding protein (RBP), besides viral RNA, IFIT5 can also bind to cellular 5′-PPP-RNA and 5′-P-RNA^15,16^, including tRNA, rRNA, snRNA, snoRNA, misc_RNA, and mRNA. Our previous study in prostate cancer was the first to demonstrate that IFIT5 could recognize and bind to certain specific pre-miRNA that had an overhang at its 5′ end, and then recruited the exoribonuclease XRN1 to degrade IFIT5-bound miRs such as miR-363, miR-101, and miR-128 with suppressive function^4^. In this study, we also demonstrate that IFIT5 can also negatively regulate the processing of miR-99a, whose precursor has a similar 5′-end overhang structure (data not shown) subjected to IFIT5 recognition and XRN1.

In cancer development, miR-99a is characterized as a tumor suppressor that is commonly down-regulated in tumor tissues^17–20^ and shown to target a variety of “oncogenic” genes, like FGFR3^21^, mTOR^22^, IGF-1R^23^, NOX4^24^, HOXA1^25^, and AGO2^26^. Functionally, miR-99a has been shown to suppresses cell proliferation in multiple cancers, including BCa, renal cancer, breast cancer, cervical cancer, esophageal squamous cell carcinoma, lung cancer, and hepatocellular carcinoma. In this study, we demonstrated the functional role of miR-99a in suppressing EMT leading to cell migration and invasion by targeting ICAM1 mRNA in BCa.

Initially, the expression of ICAM1 in bladder cancer is very low, but after BCG treatment, its expression in cancer cells and soluble ICAM1 in patient urine increased significantly^27,28^. Moreover, the level of ICAM1 in urine and serum of BCa patients is also much higher than in normal people^29–31^. Functionally, ICAM1 can interact with fibrinogen and then increase cell migration in BCa^32^, which is similar with its pro-metastatic roles in lung cancer^33^ and pancreatic ductal adenocarcinoma^34^. Besides, through interaction with LFA-1 on peripheral blood mononuclear cells, ICAM1 on HK-2 cells can accelerate EMT of HK-2 cells induced by TGF-β^35^, and the induction of EMT by monocytes infiltration in HK-2 cells is also dependent on the upregulation of ICAM1^36^.

In conclusion, this study is the first report to demonstrate the oncogenic role of IFIT5 in BCa to facilitate EMT leading to cancer invasiveness and its clinical relevance with poor survival of BCa patient. We further delineate the mechanism of action of IFIT5 to modulate tumor suppressor miR-99a in BCa. Taken together, IFIT5 represents a new therapeutic target for preventing BCa metastases and progression.

## Materials and methods

### Cell lines and cell culture

Human BCa cell lines 5637, 253J, T24, UM-UC-3, TCCSUP, and Human Embryonic Kidney (HEK) 293T cells were purchased from the American Type Culture Collection (ATCC, Manassas, VA). In all, 5637 cells were cultured in RPMI-1640 medium containing 10% fetal bovine serum (FBS), 253J, T24, UM-UC-3, TCCSUP and 293T cells were cultured in Dulbecco’s modified Eagle’s medium (DMEM) medium containing 10% FBS, all the cells were maintained at 37 °C with 5% CO_2_ in a humidified incubator. All these cell lines were authenticated with the short tandem repeat (STR) profiling periodically and Mycoplasma testing was performed by MycoAlert® kit (Lonza Walkersville, Inc. Walkersville, MD) every quarterly to ensure Mycoplasma-free.

### Bioinformatics

Gene expression data in human bladder cancer tissues were downloaded from GEO (gene expression omnibus) DataSets (GSE3167) and TCGA (the cancer genome atlas) database separately, these data files were used to analyze the expression of different genes including IFIT5, ICAM1, EMT-associated genes like CDH1 and CDH2. Also, the correlation between IFIT5 expression level and BCa patient survival was analyzed using these data.

### IHC staining and evaluation

Human primary BCa tissues were obtained from the Department of Urology, First Affiliated Hospital of Xi’an Jiaotong University, informed consent was obtained from all the patients before the tissue was collected, clinical characteristics of the patients were listed in Supplementary Table 2. Primary antibodies used in immunohistochemical (IHC) were IFIT5 (Proteintech, 13378-1-AP, 1:200), E-Cadherin (Santa Cruz, sc-8426, 1:200), and ICAM1 (Santa Cruz, sc-107, 1:200). IHC staining was performed using the EnVision System (DAKO) as our previous study described^37^. Percentage score of stained cells (1: 0–25%, 2: 25–50%, 3: 50–75%, and 4: 75–100%) and average intensity score of positive cells (0: none, 1: weak, 2: intermediate, and 3: strong) were multiplied to get the total staining scores.

### Quantitative reverse transcription-PCR and microRNA PCR array

Total RNA was isolated from cells or tissues using TRIzol reagent (Life Technologies) according to the manufacturer’s instruction. Reverse transcription was performed with miScript II RT kit (Qiagen). microRNA PCR array was performed with miScript miRNA PCR Array (Qiagen) according to the manufacturer’s instruction. Quantitative reverse transcription-PCR (qRT-PCR) was performed with miScript SYBR Green PCR Kit (Qiagen), for mRNA and pri-miRNA, 18 S was used as an internal control, for pre-miRNA and mature microRNA, RNU6B was used as an internal control. Commercial primers for miR-99a-5p, miR-99a-3p, and pre-miR-99a were purchased from Qiagen, sequences of other primers used in this study were shown in supplementary Table 3.

### Western blot

Cells were washed twice with cold PBS, and then lysed with RIPA lysis buffer containing protease inhibitor. After quantification, 30 μg total proteins were loaded into 10% SDS-PAGE gel and then blotted onto a nitrocellulose membrane. The membrane was blocked with 5% skim milk for 1 h then incubated with primary antibodies against IFIT5 (Proteintech), GAPDH (KangChen), E-cadherin, N-cadherin, vimentin, Cytokeratin-18 and ICAM1 (Santa Cruz) overnight at 4 °C. After washing with TBST for three times, the membrane was then incubated with horseradish peroxidase-conjugated secondary antibody at room temperature for 1 h. After washing with TBST for another three times, the bands were visualized by ECL system (Bio-Rad Laboratories). GAPDH was used as an internal control.

### Plasmids and cell transfecion

IFIT5 over-expression plasmid pcDNA3.1-3×Flag-IFIT5 with its control plasmid pcDNA3.1-vector, IFIT5 knock-down plasmid pLKO-shIFIT5 with its control plasmid pLKO-shcon were described in our previously study^4^. Lentivirus package plasmids pMD2.G and psPAX2 were purchased from Addgene, pre-miR-99a expression plasmid with its control plasmid miR-con were purchased from GeneCopoeia. Cell transfection was performed using jetPRIME DNA transfection reagent according to the manufacturer’s instruction. pLKO-shIFIT5 or pLKO-shcon plasmid with pMD2.G and psPAX2 plasmids were co-transfected into 293T cells together to produce lentiviral particles, stable IFIT5 knock-down or control BCa cell lines were generated after infection with these lentiviral particles and selected with puromycin. Stable IFIT5 over-expression or control BCa cell lines were generated after transfection with pcDNA3.1-3×Flag-IFIT5 or pcDNA3.1-vector plasmids and selected with G418.

### In vitro migration and invasion assay

Cell migration and invasion capabilities in vitro were determined by Transwell assay. For migration assay, cells (8 × 10^4^ for 5637, 5 × 10^4^ for 253 J, 4 × 10^4^ for UM-UC-3, 6 × 10^4^ for TCCSUP) in 200 μl serum-free medium were seeded into the upper chamber. For invasion assay, the upper chamber was coated with Matrigel for 4 h, after that, cells (1.6 × 10^5^ for 5637, 1 × 10^5^ for 253 J, 8 × 10^4^ for UM-UC-3, 1.2 × 10^5^ for TCCSUP) in 200 μl serum-free medium were seeded into the upper chamber, then 600 μl medium containing 20% FBS was added to the lower chamber. After incubation for 24 h, the upper surface of the chamber was wiped with a Q-tip, and then the Transwell was fixed with 4% paraformaldehyde, stained with 0.1% crystal violet, and washed twice with PBS. The migrated or invaded cells number was counted in five random fields.

### MTT assay

For MTT assay, 2 × 10^3^ cells in 200 µl complete medium were seeded into per well of 96-well plate, after incubation for 24, 48, and 72 h, 20 µl MTT was added to the cells, and after incubation for another 4 h in 37 °C, the medium was discarded and 150 µl DMSO was added, after 10 min of shaking, the absorbance of 560 nm was measured using the microplate autoreader (Bio-Tek Instruments).

### Dual luciferase reporter assay

pmirGLO plasmid was purchased from GenePharma. Partial of ICAM1 3′-UTR containing potential miR-99a-3p binding site was used to design wild type (WT) and mutant (MUT) pmirGLO-ICAM1 plasmid. Wild type of partial ICMA1 3′-UTR sequence was 5′-CTCCTGTTTGCATTTCACTGGGAGCTTGCACTATGCAGCTCCAGTTTCC-3′, mutant type of partial ICMA1 3′-UTR sequence was 5′-CTCCTGTTTGCATTTCACTGGCTCGAAGCACTATGCAGCTCCAGTTTCC-3′. To perform the dual luciferase reporter assay, pmirGLO-WT-ICAM1 or pmirGLO-MUT-ICAM1 plasmid with pre-miR-99a expression plasmid were co-transfected into 293 T cells together. After incubation for 24 h, luciferase assay was carried out using the dual luciferase assay kit (Promega) according to the manufacturer’s instruction.

### Subcutaneous xenograft model

Subcutaneous xenograft model was performed in male athymic nude mice, which was approved by the ethical committee of Xi’an Jiaotong University. 2 × 10^6^ UM-UC-3 subline cells or 4 × 10^6^ 5637 subline cells in 100 μl serum-free medium containing Matrigel (1:1, V/V) were injected subcutaneously into both sides of the flank region. 2 weeks after injection, xenograft tumors were harvested and the diameter was measured, tumor volume was calculated as (length × width)^2^/2^38^. After that, xenografts were subsequently used for RNA extraction, qRT-PCR and IHC.

### Statistical analysis

Statistical analyses were performed using GraphPad Prism version 5.0 software. All the data was reported as mean ± SD, differences between two groups were compared by two-tailed Student’s t-test. *P* < 0.05 was considered as statistically significant.

## Supplementary information

Supplementary infomation

Supplementary Figure 1

Supplementary Figure 2

Supplementary Figure 3

Supplementary Figure 4
