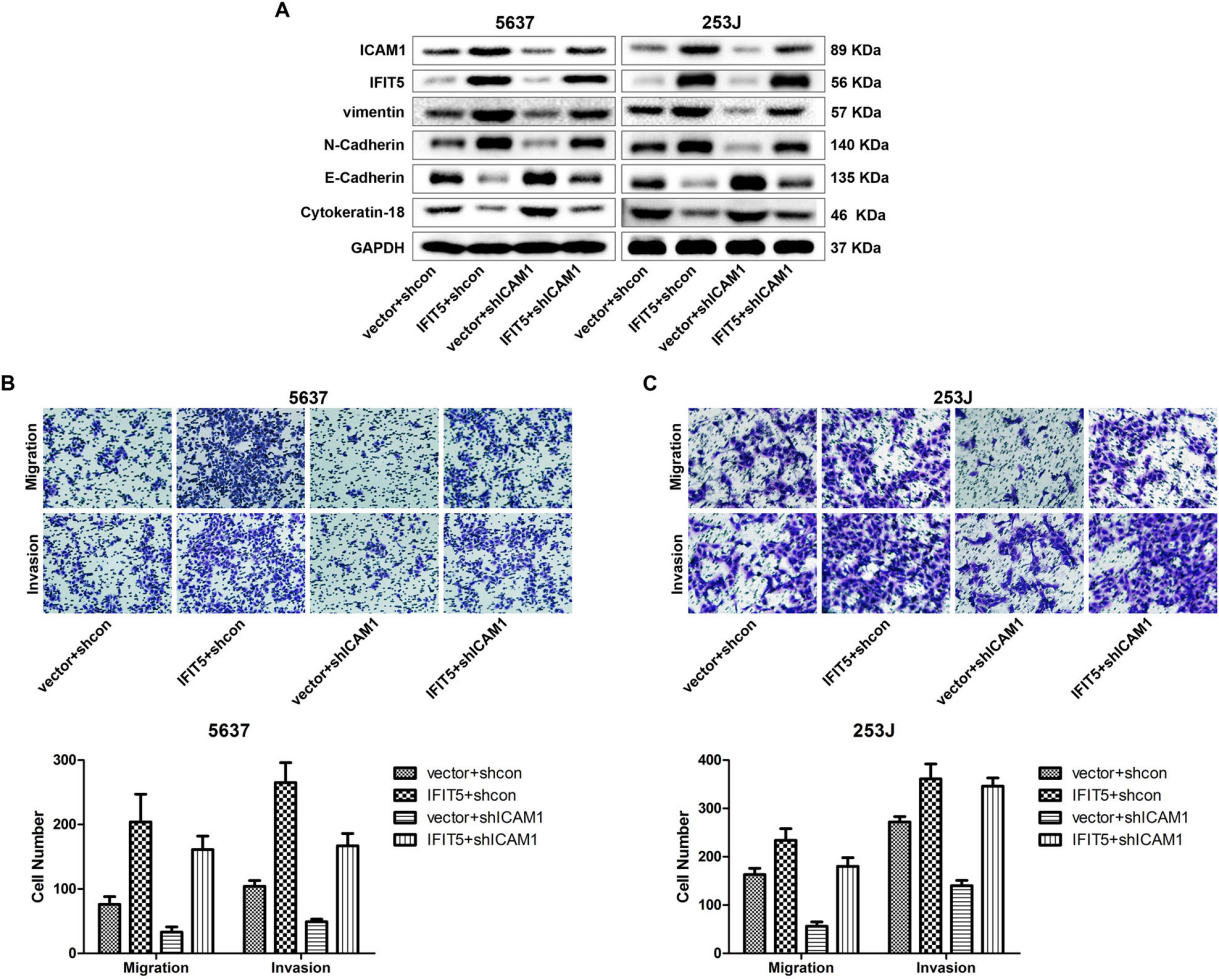# Correction: The roles and mechanism of IFIT5 in bladder cancer epithelial–mesenchymal transition and progression

**DOI:** 10.1038/s41419-025-07632-x

**Published:** 2025-04-25

**Authors:** Jun Huang, U-Ging Lo, Shiqi Wu, Bin Wang, Rey-Chen Pong, Chih-Ho Lai, Ho Lin, Dalin He, Jer-Tsong Hsieh, Kaijie Wu

**Affiliations:** 1 Department of Urology, First Affiliated Hospital of Xi’an Jiaotong University, Xi’an, P.R. China; 2 Department of Urology, The Second Xiangya Hospital, Central South University, Changsha, P.R. China; 3 Department of Urology, University of Texas Southwestern Medical Center, Dallas, TX USA; 4 Department of Microbiology and Immunology, Graduate Institute of Biomedical Sciences, College of Medicine, Chang Gung University, Taoyuan, Taiwan; 5 Department of Life Sciences, National Chung Hsing University, Taichung, Taiwan; 6 Department of Biotechnology, Kaohsiung Medical University, Kaohsiung, Taiwan

Correction to: *Cell Death & Disease* 10.1038/s41419-019-1669-z, published online 4 June 2019

Recently, we found that the second picture in panel B of Fig. 6 (the red rectangle) was misused. This has been corrected (the yellow rectangle).

Original Figure 6
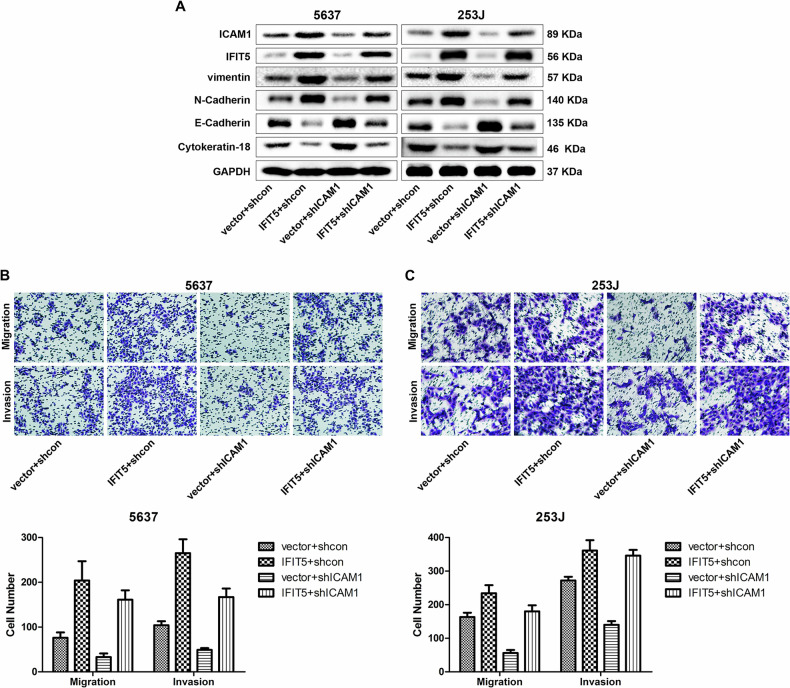


Corrected Figure 6