# Supplementary material for: The roles and mechanism of IFIT5 in bladder cancer epithelial–mesenchymal transition and progression
Source: Cell Death Dis. 2019 Jun 4;10(6):437. doi: 10.1038/s41419-019-1669-z (PMC6547745; doi:10.1038/s41419-019-1669-z)
Supplement: Supplementary file 1 — Supplementary infomation [file 41419_2019_1669_MOESM1_ESM.docx]

**Supplementary Table 1. microRNA PCR array showed the dysregulated microRNAs in IFIT5-KD UM-UC-3 cells compared to the control**

| Up-regulated microRNA | Fold change | Down-regulated microRNA | Fold change |
| --- | --- | --- | --- |
| miR-99a-5p | 7.37 | miR-3662 | 0.08 |
| miR-3163 | 3.95 | miR-143-3p | 0.10 |
| miR-7-5p | 3.53 | miR-133a | 0.14 |
| miR-135a-5p | 2.88 | miR-9-3p | 0.18 |
| miR-27b-3p | 2.75 | miR-296-5p | 0.23 |
| miR-100-5p | 2.63 | miR-181a-5p | 0.52 |
| miR-194-5p | 2.60 | miR-223-3p | 0.54 |
| miR-125b-5p | 2.58 | miR-29b-3p | 0.55 |
| miR-15b-5p | 2.53 | miR-616-3p | 0.55 |
| miR-27a-3p | 2.50 | miR-375 | 0.58 |

**Supplementary Table 2. Clinical characteristic of the patients**

|  | NMIBC | MIBC |
| --- | --- | --- |
| Total patient number | 30 | 30 |
| Age (years, M±SD) | 58.8±10.4 | 63.9±9.5 |
| Gender (%) |  |  |
| Male | 25（83.3%） | 27（90.0%） |
| Female | 5（16.7%） | 3（10.0%） |

**Supplementary Table 3. Sequences of primers used in this study**

| Primer | Forward (5’-3’) | Reverse (5’-3’) |
| --- | --- | --- |
| 18S | GGAATTGACGGAAGGGCACCACC | GTGCAGCCCCGGACATCTAAGG |
| IFIT5 | TAAAAAAGGCCTTGGAGGTG | CCAGGTCTGTGTAGGCAAAT |
| ICAM1 | GGCCTCAGTCAGTGTGA | AACCCCATTCAGCGTCA |
| pri-miR-99a | ATTAATAGGGGGCCCATGCAA | ATTGTTGAACGGCACTGTGT |

**Figure Legends**

**Supplementary Figure 1. Neither OE nor KD of IFIT5 affects BCa cell proliferation.** (A-B) no significant changes of cell proliferation after OE of IFIT5 in 5637 and 253J cells. (C-D) no significant changes of cell proliferation after KD of IFIT5 in TCCSUP and UM-UC-3 cells.

**Supplementary Figure 2. IFIT5-induced microRNA expression in BCa cells**. (A) microRNA expression changes after OE of IFIT5 in 253J cells. (B) microRNA expression changes after KD of IFIT5 in TCCSUP cells.

**Supplementary Figure 3. ICAM1 expression in BCa subcutaneous xenografts.** (A) ICAM1 expression was higher in IFIT5-OE 5637 subcutaneous xenografts. (B) ICAM1 expression was lower in IFIT5-KD UM-UC-3 subcutaneous xenografts.

**Supplementary Figure 4. Data from TCGA shows no differences in RFS (A) and OS (B) between higher IFIT5/lower pre-miR-99a and lower IFIT5/higher pre-miR-99a.**
